# The complete mitogenome of one commercial *Saccharina* cultivar in Chinese market and its evaluation of genetic relationship

**DOI:** 10.1080/23802359.2018.1473736

**Published:** 2018-05-15

**Authors:** Xiangxiang Liu, Jun Li, Chuangang Chang, Jing Zhang

**Affiliations:** a Qilu University of Technology (Shandong Academy of Sciences), Jinan, Shandong Province, People’s Republic of China;; b Shandong International Fisheries Corp, Jinan, Shandong Province, People’s Republic of China

**Keywords:** Commercial Saccharina cultivar, genetic relationship, mitogenome, parental origin

## Abstract

Here, the complete mitogenome of one commercial *Saccharina* cultivar in Chinese market which marked ‘Shichang’ was reported. Circular-mapping mitogenome with an overall A + T content of 64.70% was 37,657 bp in size. It encoded three rRNAs (23s, 16s, and 5s), 25 tRNAs, 38 proteins (including three open reading frames, ORFs). Gene component and arrangement of ‘Shichang’ mitogenome were identical to those reported *Saccharina* cultivars. Further phylogenetic analysis of mitogenomes confirmed that ‘Shichang’ had closer relationship with cultivar ‘Pingbancai’, which suggested its parental origin and genetic relationship.

*Saccharina* (Laminariales, Phaeophyceae) is one of the most important seaweeds with its great economic value (Kain 1979). In China, about 20 cultivars have been bred (Zhang et al. 2016b), and most of them were selected from *Saccharina japonica*. However, parental origin of *Saccharina* cultivar in Chinese market is not clear due to the genetic hybrid during the breeding and cultivation. Here, we obtained the complete mitogenome of one commercial cultivar from the market of Weihai, Shandong, China (37°16′N, 122°41′E) and conducted phylogenetic analysis to provide new molecular data to evaluate the parental origin and genetic relationship of Chinese commercial *Saccharina* cultivar.

‘Shichang’ sample (specimen number: 201705002) was stored at –80 °C for DNA extraction. The protocol and data processing of this work were followed by Zhang et al. (2011).

The complete mitogenome of ‘Shichang’ was characterized as a circular molecule of 37,657 bp (GenBank accession number MG712781). The nucleotide contained as follows: A = 10,700 (28.4%), T = 13,664 (36.29%), G = 7750 (20.58%), and C = 5543 (14.72%). The mitogenome had an overall A + T content of 64.70%, exhibiting a high AT richness. Cumulative AT-skew (–0.1218) and GC-skew (0.1660) analysis of mitogenome reflected a slight bias towards T and G in nucleotide composition on H-strand. The mitogenome encoded 66 genes including three rRNAs, 25 tRNAs, 35 protein-encoding genes, and three open reading frames (ORFs). Apart from *rpl*2, *rpl*16, *rps*3, *rps*19, *tac*C, and ORF130, 60 genes were encoded on H-strand. In this study, *rps8–rpl6–rps2–rps4* was also found as a conserved gene cluster in those reported *Saccharina* species and cultivars. All protein-encoding genes used ATG as the initial codon and 68.42% used TAA as stop codon, higher than that for TAG (21.05%) and TGA (10.53%).

According to the total alignment of ‘Shichang’ and *S. japonica*, only 11 nucleotide mutations were detected and four led to the variety of amino acid. The result of this work indicated the component and arrangement of *Saccharina* species and cultivars mitogenomes had highly conservative evolution (Yotsukura et al. 2010; Zhang et al. 2011, 2013, 2016a, 2017).

Bayesian method based on the whole mitogenome sequences of 17 available *Saccharina* and *Laminaria* algae was used to reconstruct the phylogenetic tree (Figure 1). *Ectocarpus siliculosus* was selected as the outgroup. The results of phylogenetic analyses showed that all algae were divided into two groups: *Saccharina* and *Laminaria*. ‘Shichang’ together with other reported *Saccharina* cultivars were all in *Saccharina* clade and ‘Shichang’ had the closest relationship with cultivar ‘Pingbancai’. This study would facilitate us to better understand the parental origin and genetic relationship of commercial *Saccharina* cultivar in Chinese market.

**Figure 1.**
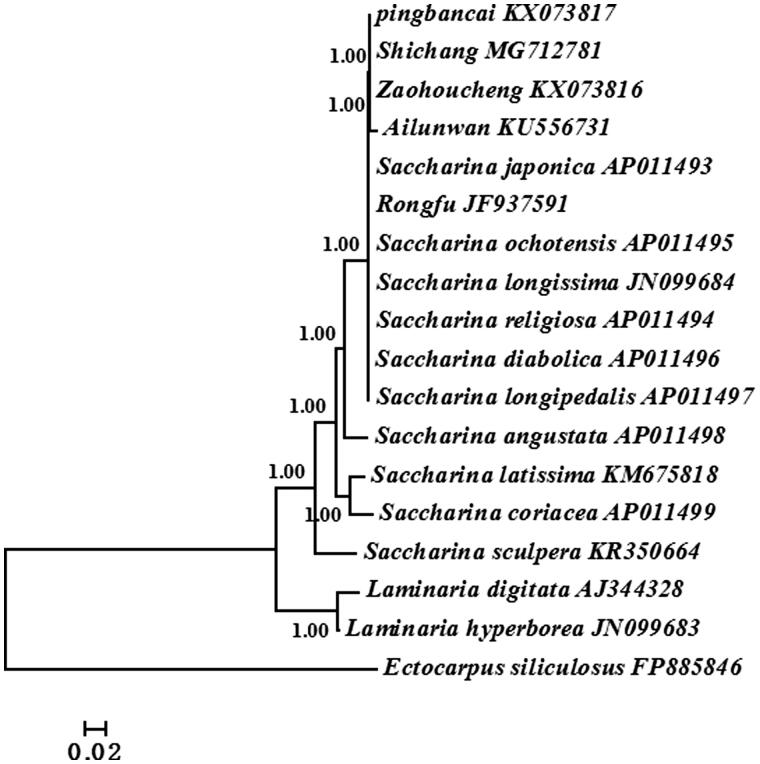
Phylogenetic trees derived from Bayesian analysis constructed based on concatenated nucleotide sequences of 35 mtDNA protein-encoding genes.
